# Superposition-free comparison and clustering of antibody binding sites: implications for the prediction of the nature of their antigen

**DOI:** 10.1038/srep45053

**Published:** 2017-03-24

**Authors:** Lorenzo Di Rienzo, Edoardo Milanetti, Rosalba Lepore, Pier Paolo Olimpieri, Anna Tramontano

**Affiliations:** 1 Department of Physics, Sapienza University, Piazzale Aldo Moro 5, 00184, Rome, Italy; 2 Istituto Pasteur-Fondazione Cenci Bolognetti, Viale Regina Elena 291, 00161, Rome, Italy

## Abstract

We describe here a superposition free method for comparing the surfaces of antibody binding sites based on the Zernike moments and show that they can be used to quickly compare and cluster sets of antibodies. The clusters provide information about the nature of the bound antigen that, when combined with a method for predicting the number of direct antibody antigen contacts, allows the discrimination between protein and non-protein binding antibodies with an accuracy of 76%. This is of relevance in several aspects of antibody science, for example to select the framework to be used for a combinatorial antibody library.

The comparison of protein structures has had a major impact in our understanding of protein structure and function and has opened the road to a number of methods for their classification and structure prediction, often useful for the inference of their function.

Homologous proteins share a common core, the extent and similarity of which depends on their evolutionary distance^1^. This has allowed the development of relevant and widely used methods such as comparative modeling^2^. With this strategy, when one or more structures of the protein of an evolutionary family are known, the conserved regions can be used as a template for modeling the corresponding regions of proteins of unknown structure from the same family^1^.

The structurally divergent regions clearly cannot be predicted with this approach. They are nevertheless extremely relevant because in some cases the differences among related structures might be directly linked to their specificity, i.e. to their specific binding partner(s)^3^. The comparison of binding sites among unrelated proteins, therefore, can help identifying similarities related to their function^4^.

Here we concentrate on a specific, and relevant, class of proteins, i.e. antibodies. These molecules have a very conserved structural framework and their specificity is, as expected, determined by the surface of their binding site brought about by regions that are appropriately named “hypervariable loops”^5^,^6^,^7^ or Complementary Determining Regions (CDRs). The definition of hypervariable loops and CDRs do not perfectly overlap^8^. Here we will use both terms interchangeably. The structure of the main chain of five of these loops can be predicted quite accurately by taking into account the position and identity of a few specific key amino acids, according to the so-called canonical structure method^5^,^7^. More recently an effective method for the prediction of the sixth loop (named H3) has also been developed^9^ and therefore prediction of the antigen binding site structure has been improved^10^.

The next challenge is to relate the structure of the binding site to its function, i.e. to the nature of the bound antigen and indeed, in the past years, there have been several attempts in this direction^11^,^12^,^13^.

For example, Collis *et al*.^11^ analyzed the correlation between the lengths of the antigen binding loops and the nature of the antigen concluding that preferences for certain combinations are related to the nature of the antigen. They also looked at the effect of the sequence composition of the antigen binding site and observed that alanine is under-represented only in hapten binders, while hapten, protein and virus binders have a lower than expected frequency of phenylalanine. Glycine and valine are instead under-represented in all classes except peptide binders. These rules are useful as general guidance, for example in antibody design, but do not seem to reach an accuracy above 50% when used as features in automatic learning methods^11^.

Also Raghunathan *et al*.^12^ performed a careful analysis of known antibody structures (140 cases) in complex with proteins (55), peptides (39) and haptens (46). They found several interesting structural properties of the interface region for the known complexes, highlighting preferences in the nature and location of the antibody antigen contacts in the various classes. Still, they could only detect weak relationships between the sequence and structure of the antigen binding site and the nature of the antigen. For example, they showed that anti-peptide and anti-hapten antibodies predominantly have longer L1 loops (11–13 residues), while short L1 loops are found in protein binding antibodies, and there seems to be no signal in the length of the other loops.

Lee *et al*.^13^ used a geomorphic description for the shape of the antibody binding sites and classified them in five classes, depending on their shapes: Cave, Crater, Canyon, Valley, Plain. They report some correlation between the shape and the antigen type in a dataset of 229 antibody structures. For example, hapten antigens mostly bind to the cave type antibodies (43.1% of the cases) and there is a preference of peptide/carbohydrate/nucleic acid antigens for crater type antibodies (45.5% of the cases). Proteins often (43.3% of the times) bind to the plain type antibodies.

To the best of our knowledge, an effective method to accurately classify the type of antigen bound by an antibody on the basis of its binding site sequence and structure is still missing. This is certainly due to the inherent difficulty of the problem, but the problem is worsened by the biased composition of available datasets that contain mostly protein binding antibodies and very few other types. There is no doubt that an effective method for detecting the similarity in shape of the binding site is an essential ingredient to progress in this direction^13^ and that it is important to be able to compare the actual surface rather than the atomic coordinates of the binding sites since the loops forming the binding site can have different length and structure and yet result in a similar binding surface. Furthermore, the structural comparison of binding site surfaces can allow the analysis of antibodies produced by patients of B-cell malignancies with implications for their etiology and prognosis^14^,^15^.

Here we illustrate a protocol to quickly compare the surface of antibody binding sites that is superposition free, rotation and translation invariant and sufficiently fast to allow the comparison of a given antibody binding-site with those of a large dataset.

The protocol uses a moment-based representation of the binding site. Moment-based representations are a class of mathematical descriptors of shape, originally developed for pattern recognition^16^ whereby a structure is described by a quantitative numerical representation that can be expanded as a series of orthogonal polynomials. The description can be as detailed as desired according to the selected limit on the order of the expansion used^17^. In particular we use here a method based on the 3D Zernike descriptor formalism^18^ tailored to compare antibody binding sites.

Zernike polynomials were first introduced by Zernike^19^ and have proven to be very accurate in image retrieval. Canterakis generalized them to a 3D space^20^. Next, Novotni and Klein adapted it to shape retrieval^21^. The Zernike description has already been effectively used in structural biology for a wide range of purposes, such as, for example, protein tertiary structure retrieval and comparison^22^, protein-protein docking^23^ and shape-based ligand similarity searching^24^.

We show here that a classification based on this approach can improve the prediction of the nature of the antigen recognized by an antibody, given the coordinates of its antigen binding site.

## Results

### The procedure

**Figure 1:**
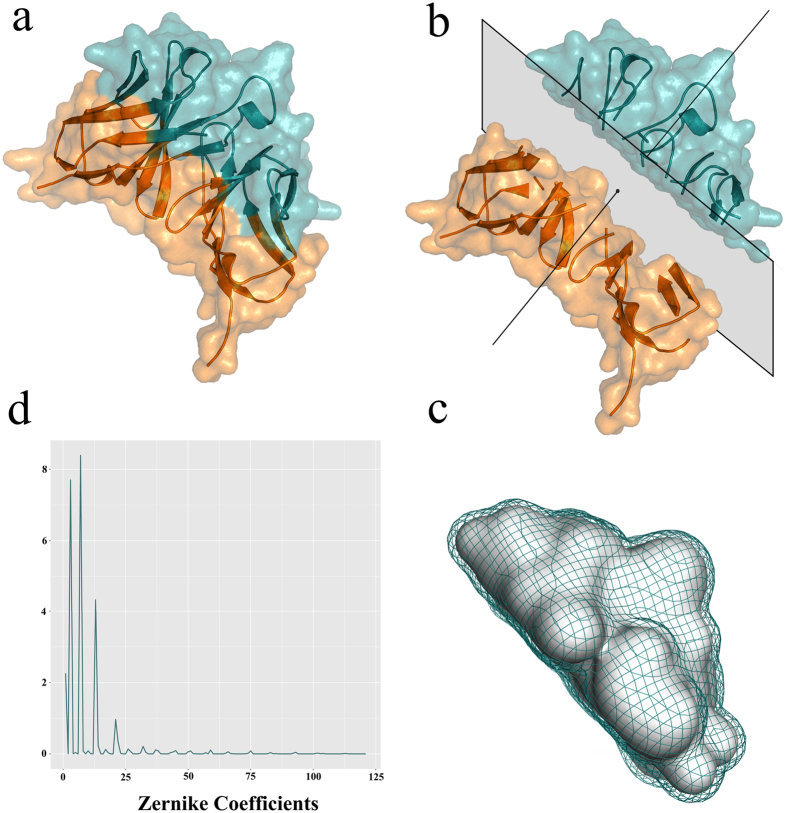
Schematic representation of the steps involved in the Zernike description of the binding site surface. (**a**) Identify the binding site (cyan) of the antibody. (**b**) Draw a central axis (blue line) and the plane that divides the antibody binding site from the framework regions (orange) as described in Methods. (**c**) Voxelize the Gaussian surface of the subspace containing the binding site. (**d**) Compute the 121 invariant Zernike descriptors of the surface.

The overall scheme of our procedure is shown in Fig. 1.

The first step in our protocol is the definition of a sub-space containing the binding site of an antibody so that we can quantitatively describe its surface. After renumbering the antibodies according to the Chothia numbering scheme^5^,^6^, we identify, for each antibody, a straight line (r) connecting the centroid of the c-alpha coordinates of a set of very conserved residues at the interface between the light and heavy chain, namely H:22, H:103, L:23, L:98 and the geometric center of all c-alpha atoms belonging to the binding site. We next project the coordinates of each atom of the binding site on the r line obtaining a new coordinate, s, defined along the r direction, and compute its minimum (s_min) and maximum (s_max) values. The binding site is formed by all atoms with an r coordinate between s_min and s_max. We subsequently define a plane perpendicular to r that passes through s_min. All atoms included in the r > 0 subspace defined by this plane are part of the binding site surface. The binding site surface also contains the “artificial” surface defined by the separating plane, which, in the protein structure, is not accessible to the solvent, however this “artificial” surface is very similar among different antibodies and therefore does not influence the subsequent analyses (see Fig. 1).

The next step is the calculation of the surface of the region included in the sub-space, i.e. the binding site plus that formed by the artificial plane. We use the Gaussian Description^25^: each atom is approximated by a Gaussian density distribution. The parameters of each Gaussian are chosen so that 95% of the Gaussian volume falls within the Van der Waals radius of the corresponding atom^26^. The overall analytical description of the density is computed using the coalescence theorem and can be easily integrated^25^.

The subsequent step consists in voxelization. We scale the molecular surface in the unit sphere and place it in a Cartesian grid of dimensions 64^3^. We associate the value of the integral of the molecular shape density computed over the volume of the voxel to each voxel.

The surface of interest can therefore be expressed as a function f(r, θ, ψ) in any arbitrary polar reference system and can be expanded as a series of polynomials with appropriate coefficients. In particular, if the latter are the Zernike coefficients (see Methods), their norm (3DZD) is a description of the surface that is invariant for both translation and rotation.

We computed the Zernike descriptors 3DZD for the binding sites of all the antibodies in our dataset composed of 329 antibodies of known structure (see Methods) and clustered them as described in Methods. For comparison, we also clustered the same binding sites using a coordinate distance-based measure, i.e. the TM score^27^.

**Figure 2:**
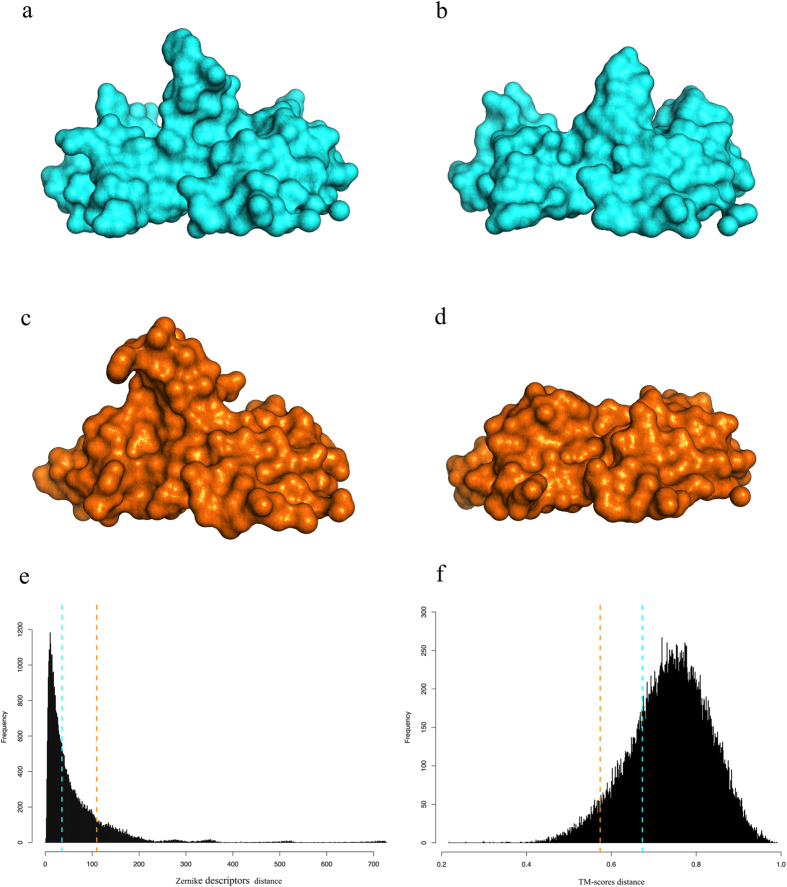
Surface comparison of two pairs of binding sites. (**a**,**b**) are two antibodies (cyan) with their Van der Waals surfaces deemed to be similar by visual inspection (PDB codes 2ny7 and 3vg9); (**c**,**d**) are two antibodies (orange colored) with Van der Waals surfaces considered different by visual inspection (PDB codes 3lev and 4g6m); Distributions of all the antibody-antibody distances calculated using the Zernike (**e**) and the TM method (**f**). The distance values of the two pairs are shown in both distributions (Zernike and TM) by dashed lines of the same color as the corresponding antibody surfaces.

A similarity in Zernike moments is much more informative about the actual shape similarity of a protein region, as it can be visually appreciated by the example in Fig. 2 where two pairs of antibody binding site surfaces are compared. Those in the top panel are close in the Zernike classification (49^th^ percentile) and far in TM-score (85^th^ percentile), while those in the central panel are close in TM-score classification (5^th^ percentile) and far in Zernike (23^rd^ percentile).

### Clustering

We clustered all the antibody binding sites of our dataset using the Ward method as linkage function while the Manhattan distance among the Zernike descriptors (Zernike cluster) and the TM score computed over the main chain atoms of the binding site (TM cluster) were used as distance metrics. We also tested different metrics and clustering methods obtaining very similar results (data not shown).

**Figure 3:**
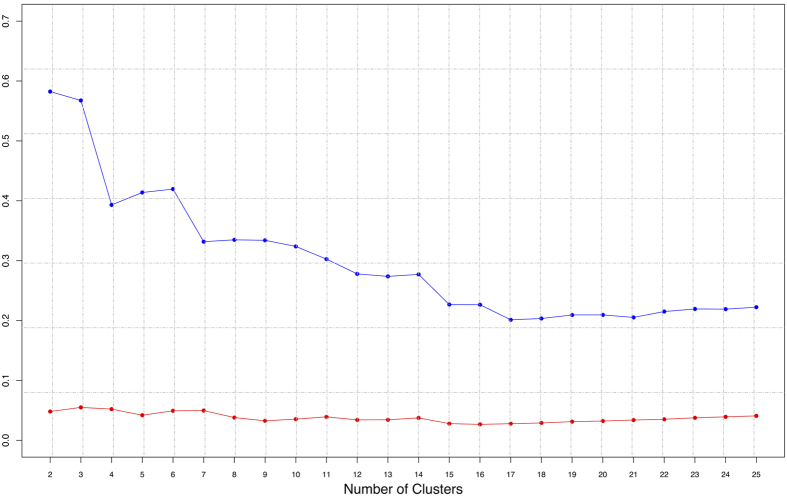
The average silhouette value as a function of the number of clusters. The Silhouette values of the Zernike and TM-based clusters are shown in blue and red, respectively.

The optimal clustering cut was estimated using the silhouette parameter^28^ varying the number of clusters from 2 to 25 (Fig. 3, blue dots).

For the Zernike cluster, there is a clear maximum for the silhouette value for two clusters and we call these groups “cluster 1” and “cluster 2”.

The results of the Silhouette analysis on the TM cluster are much less informative (Fig. 3, red dots). To be able to compare the results, we selected to divide in two clusters also the classification obtained from the TM-score based analysis.

**Figure 4:**
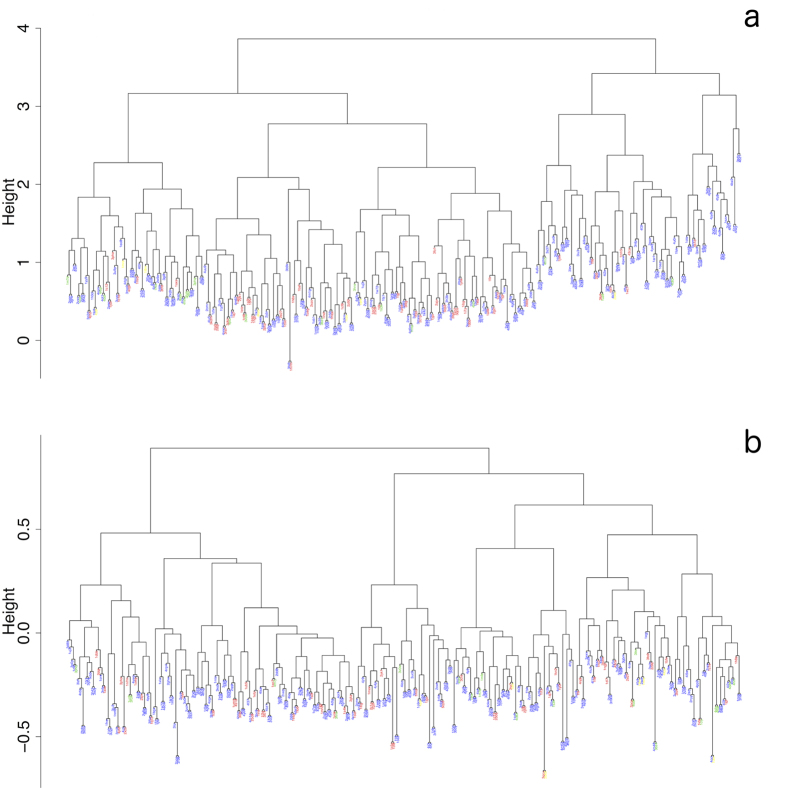
Clustering of antibody binding sites. The figure reports the clustering based on the Zernike invariant descriptors (**a**) and the TM-score (**b**). In both clusters the antibody PDB IDs are colored according to the biological type of their cognate antigens. Protein binding are indicated in blue, hapten binding in red, carbohydrate binding in green and nucleic acids binding in yellow. The clustering was performed using the Ward distances reported in logarithmic scale on the y-axis.

In Fig. 4 we show the results of the Zernike and TM clustering applied to the whole antibody binding site dataset. We labeled and colored each antibody PDB ID according to the type of its cognate antigen (proteins, haptens, carbohydrates, nucleic acids).

**Figure 5:**
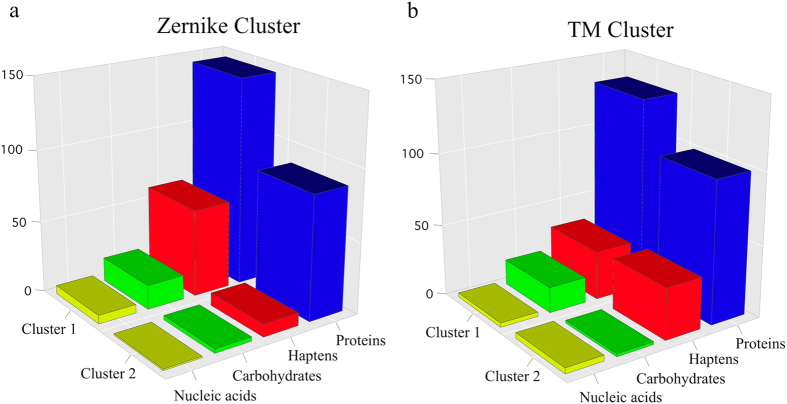
Histograms of the cluster population. Number of antibody binding sites belonging to the clusters based on the Zernike invariant descriptors (**a**) and the TM-score (**b**) shown in Fig. 4. Cluster elements are grouped according to the type of recognized antigen and colored using the same coloring scheme as in Fig. 4.

The Zernike cluster provides a representation of the binding site shape that better correlates with the specific type of antigen. Indeed, the second cluster, containing 100 binding sites, includes almost exclusively protein binding antibodies (87). This is not the case for the TM based clusters, both of which include approximately the same percentage of antibodies that bind proteins and that do not (Fig. 5).

The first cluster is more promiscuous, both in the Zernike and in the TM clusters, and is not enriched in any statistical significant way by antibodies that bind a specific type of antigen.

The question arises about what makes the more promiscuous antibodies of the first cluster different from the more specific antibodies of the second cluster.

**Figure 6:**
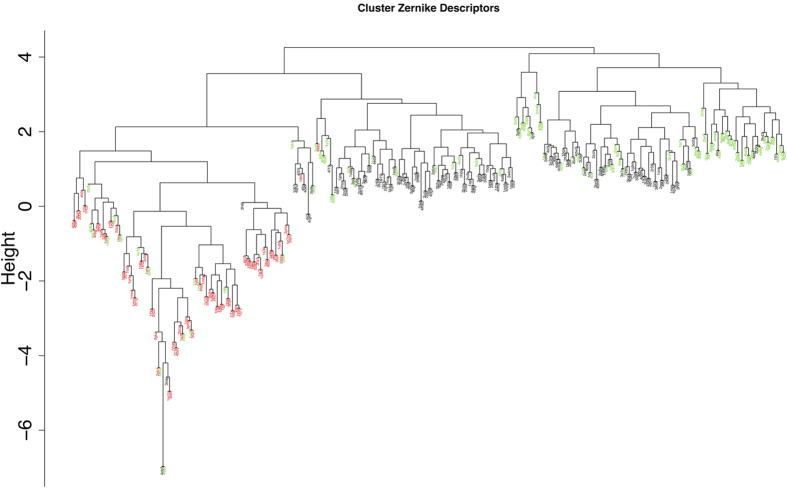
Clustering of the antigens of the dataset. Clustering of the antigens using their Zernike descriptors (see Methods). All the non-protein antigens are shown in red. Proteins bound by antibodies belonging to cluster 2 of Fig. 4-a are shown in green. The remaining antigens are colored in black.

The cluster separation is not due to trivial factors, such as the size of the antigen (Supplementary Figure 1), but it seems related to the shape of the antigen itself. Indeed, if we cluster all the antigens of our antibody dataset according to their overall Zernike descriptors, those bound by antibodies belonging to the second cluster are more similar in shape among them than to the proteins bound by antibodies of the first cluster (78% of the proteins bound by antibodies of the second cluster are close together in terms of their overall shape) (Fig. 6).

**Figure 7:**
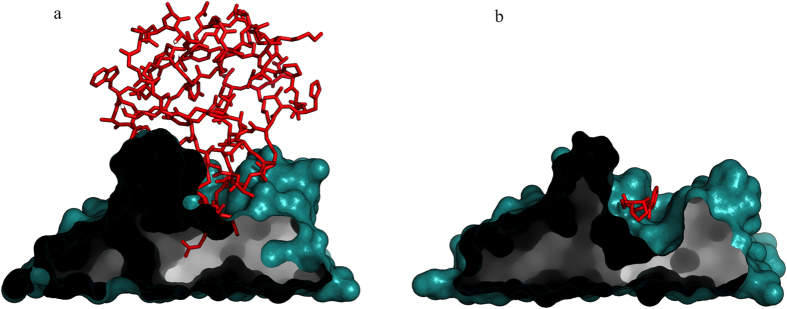
Similar antibody binding sites recognize different antigens. The figure shows two antibodies belonging to cluster 1: (**a**) an hapten binding antibody (PDB ID: 4AEI) and (**b**) a protein binding antibody (PDB ID: 1Q72).

The antibodies of the first cluster bind both haptens and proteins, but visual analysis shows that in the latter case they recognize a protruding part of the protein surface that is inserted in the binding site and resembles very much the mode of binding of an hapten or, in general, of a small molecule, as it can be appreciated by the example shown in Fig. 7. However, in these cases, we expect the number of contacts between the antibody and its antigen to be different.

We therefore devised a strategy that combines the Zernike clustering with a method that predicts with good accuracy the probability that a given antibody amino acid is in direct contact with the antigen. The method, named proABC^29^ (prediction of antibody contacts), is based on automatic learning, random forest in particular, and has been trained using all the antibodies of known structure in complex with an antigen. To ensure an unbiased result, we retrained the random forest 329 times, each time excluding one of the antibodies from our dataset and predicting the probability that its amino acids are in contact with the antigen. We selected a probability threshold of 50%, in other words we assumed that amino acids predicted to be in contact with the antigen with a probability greater than 50% are counted as contacts.

**Figure 8:**
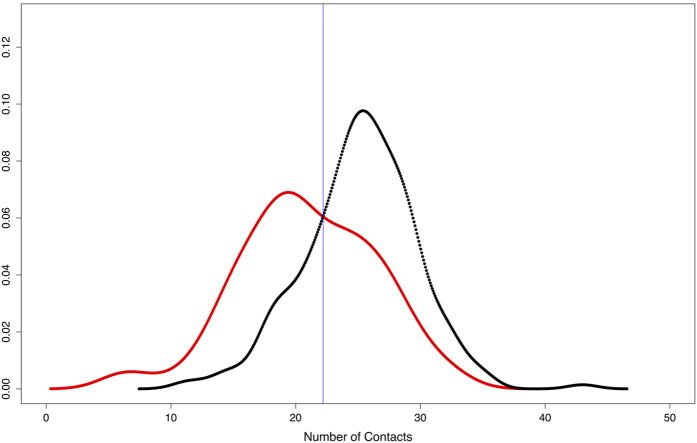
Distributions of the predicted number of contacts. The distribution of the number of predicted contact for non-protein and protein binding antibodies are shown in red and black, respectively. The blue line indicates the selected threshold value (i.e. 22).

As it can be seen from Fig. 8 the distributions of the predicted number of contacts between the antigen and the antibody for protein and non-protein binding antibodies are different and cross each other at a value of 22. 75% of the protein binding antibodies have a predicted number of contacts higher than this value.

We next performed the following steps: given an antibody structure, we compute the Zernike moments of its binding site and its distance with the median values of the two clusters. If the antibody is closer to the median of the second cluster we predict it to be protein binding. In the other case, we predict the number of contacts using proABC. If the number of predicted contacts is above 22, we predict the antibody to be protein binding.

The overall accuracy of the method is 76%. We correctly assign the antigen type to 249 out of 329 antibody structures. In 33 cases we classify protein binders as non-protein binders, the opposite is true for the remaining 47. It is worth mentioning that the first step of the procedure (i.e. classifying an antibody as protein binding if its surface belongs to cluster (2) only misclassifies 10 antibodies out of 100. Out of the 229 antibodies belonging to cluster 1, we incorrectly classify 70 antibodies. Interestingly, if instead of using the predicted number of contacts we used the actual number of contacts, the number of incorrect predictions would decrease to 16, thus suggesting that the main cause of the classification error is the accuracy of the contact prediction.

We also carefully analyzed the structure of the other misclassified examples, but we could not detect any specific feature that could be correlated to their incorrect prediction. The list of the PDB ID of antibodies incorrectly classified is shown in Supplementary Material.

## Discussion

In most cases, the ultimate goal of the analysis of antibody structures is to try and deduce information about the bound antigen, an aspect that is of relevance for several applications, from the selection of appropriate antibodies as a starting point for engineering experiments to the identification of clinically relevant properties of antibodies detected in B-cell malignancies.

However, the identification of the type of molecule bound by an antibody is a very complex and so far unsolved problem. While the antigen binding site of the antibody is complementary in shape to its cognate epitope, the latter can be of many different shapes even in similar molecules and, on the contrary, be similar in different molecules or types of molecules.

Here we presented a method, based on the Zernike descriptors of surfaces and on the prediction of the number of contacts between the antibody and its antigen, that can effectively provide information about the shape of the epitope and predict with reasonable accuracy whether the antibody binds a protein molecule.

It should be mentioned that our approach for the surface comparison of antibody binding sites offers a significant technical advantage over other approaches since it is superposition free. Furthermore, visual inspection clearly shows that the surface similarity is better detected using the Zernike descriptors than using methods relying on the comparison of the coordinates of the atoms of the binding sites.

Even though, as stated before, there is not a very strong relationship between the shape of the paratope and the type of antigen, we have shown here that, by combining a Zernike-based classification of the binding site and an estimate of the number of contacts, we can predict, given the structure of an antibody, whether it does bind a protein with an overall accuracy of 76%, with the misclassification error being mostly due to the limited accuracy of the contact prediction method.

This result, in conjunction with the present availability of methods for the accurate prediction of antibody structure, represents a step towards the very elusive, but important, goal of predicting antibody specificity. In practice, it can also guide the design of antibody libraries and perhaps help formulating hypotheses about the specificity of the antibodies produced by malignant B-cells^15^.

The available dataset of antibody structures with a non-protein bound antigen is very limited and this makes it extremely difficult to also attempt to predict the specific nature of the antigen as opposed to the binary distinction between protein and non-protein binding ones. We are confident that with an increase in the available complex structures, this method, possibly in conjunction with other tools, might help relate more precisely the shape of the antigen binding site to the chemical nature of the cognate antigen.

## Methods

### Dataset

We selected 329 antibody complexes with a level of redundancy lower than 90%, using the database SabDab DB^30^, solved with a resolution better than 3 Å. 232 of them bind a protein antigen, 70 bind haptens, 20 bind carbohydrates and 7 nucleic acids. The dataset is available at www.biocomputing.it/Abzern.

We renumbered the sequence of each antibody according to the Chothia numbering scheme and selected the sub-space containing the binding site using in house R scripts relying on the “bio3d”^31^ and “geometry” packages^32^ of R.

### The Zernike moments

The function (*r, θ, φ*) describing the surface of the sub-space containing the binding site of an antibody, defined as described above, can be represented by a series expansion in an orthonormal sequence of polynomials:


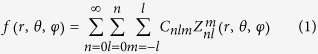


where 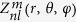 are the 3D Zernike polynomials and *C*_*nlm*_ are the Zernike moments defined below. The indices n, m and l are integers and are called order degree and repetition, respectively.

The equality in the expression only holds when the sum over n goes to ∞, but it can be truncated at the desired level of approximation at the expense of describing the surface at different levels of details. In our analysis, the series is truncated at n = 20 and therefore we deal with 121 descriptors for each binding site.

The Zernike polynomials can be written as:


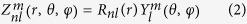


where R only depends on the radius r, it has been defined Canterakis^20^ and is given by:


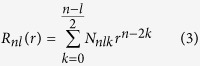


where N is a normalization factor.

The Y functions are complex Spherical Harmonics depending on both θ and ϕ (see Supplementary Material).

The 3D Zernike moments of *f(r, θ, φ*) are defined as the coefficients of the expansion of *f(r, θ, φ*) in the Zernike polynomial basis, i.e.:


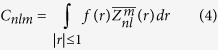


where 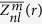 is the complex conjugate of the polynomial.

As these moments are not invariant under rotation, in order to obtain transformation invariant descriptors, i.e. the 3D Zernike Descriptors (3DZD), the norm of these vectors must be computed:


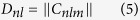


Details about the derivation of the above representation can be found in^17^,^18^.

The 3D Zernike polynomials are defined within the unit sphere, so it is necessary to appropriately scale the *f(r, θ, φ*) function. As suggested by^26^, we selected to scale the function so that all the binding site region falls within 60% of the unit sphere. The calculation of the Gaussian surface, the voxelization and the computation of the Zernike coefficients are made using the python code described in ref. 26.

### Statistical analysis

Each binding site is described by a 121 dimensional vector in the Zernike description. We clustered the descriptors of the binding sites using the Manhattan distance and the Ward method as linkage function^33^ via the “hclust” function of the “Stats” package of R^34^.

TM score was calculated using a in-house perl script and in a all-against-all manner^27^, after superposition of the antibody structures using the LGA package^35^. The Ward method has been used for clustering TM distances (defined as 1-TM score). The silhouette values for all clusters were computed using the silhouette function of the “Cluster” package of R.

The 3D images of the molecular surfaces were generated using Pymol^36^.

### Software availability

The script to compute the Zernike moments of the antigen binding site of an antibody is available for download at www.biocomputing.it/Abzern. It is written in Python and only requires a local installation of the R package. The readme file in the same location describes how to run the code. The user needs to provide a folder/directory containing any number of PDB structures of antibodies in PDB format^37^ and the desired order of expansion of the Zernike series (defaults to 20). Starting from the antibody structure coordinates, the tool identifies the light and heavy chains of the antibodies, renumbers them according to the Chothia numbering scheme, identifies the binding site, computes the corresponding Zernike descriptors and outputs them as a comma separated CSV file.

## Additional Information

**How to cite this article:** Di Rienzo, L. *et al*. Superposition-free comparison and clustering of antibody binding sites: implications for the prediction of the nature of their antigen. *Sci. Rep.* **7**, 45053; doi: 10.1038/srep45053 (2017).

**Publisher's note:** Springer Nature remains neutral with regard to jurisdictional claims in published maps and institutional affiliations.

## Supplementary Material

Supplementary Information
