# Supplementary material for: Superposition-free comparison and clustering of antibody binding sites: implications for the prediction of the nature of their antigen
Source: Sci Rep. 2017 Mar 24;7:45053. doi: 10.1038/srep45053 (PMC5364466; doi:10.1038/srep45053)
Supplement: Supplementary Information [file srep45053-s1.pdf]

Superposition-free comparison and clustering of antibody binding sites: implications for the prediction of the nature of their antigen

Lorenzo Di Rienzo[1,#], Edoardo Milanetti [1,#], Rosalba Lepore [1,2], Pier Paolo Olimpieri[1, *] and Anna Tramontano[1,2]

[1] Department of Physics, Sapienza University, Piazzale Aldo Moro 5, 00184 Rome, Italy

[2] Istituto Pasteur-Fondazione Cenci Bolognetti, Viale Regina Elena 291, 00161 Rome, Italy

[*]To whom correspondence should be addressed: Pierpaolo.Olimpieri@uniroma1.it

[#] These authors contributed equally

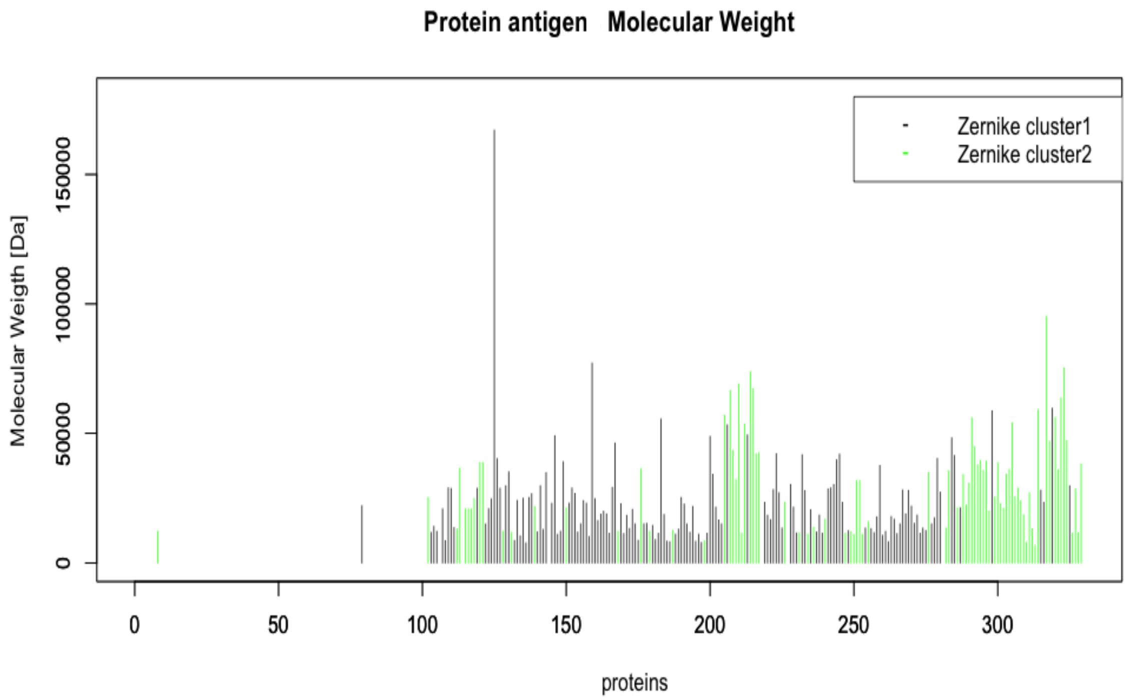

Supplementary Figure 1

Molecular weight distribution for the protein antigens of the dataset.

The antigens are reported in the same order as they appear in the dendogram of Figure 6. All the proteins bound to antibodies belonging to cluster 2 of Figure 4-a are shown in green, the others in black.

Spherical Harmonics

Spherical harmonics are functions defined on the surface of a unit sphere that are often useful in solving partial differential equation. They constitute an orthogonal and complete set of functions, and thus they may be used as a base for series expansions in the domain defined by the surface of that sphere. In spherical coordinates they are defined by:

$$Y_l^m(\theta,\varphi)=(-1)^{\frac{m+|m|}{2}}\left\{\frac{2l+1}{4\pi}\frac{(l-|m|)!}{(l+|m|)!}\right\}^{1/2}P_l^{|m|}(\cos\theta)e^{im\varphi} = N_l^m P_l^{|m|}(\cos\theta)e^{im\varphi}$$

in which it the dependence on the longitudinal angle θ and the azimuthal angle φ is included, $P_l^{|m|}$ are the Legendre polynomials; we introduce now the invariance properties of these functions.

Invariance of spherical harmonics

It is possible to demonstrate that the norm of the projections of a generic function f (i.e. the output function of the voxelization step) along the spherical harmonics is invariant under rotation.

In order to do this, we define a vector Y_l called *spherical harmonics vector* given by:

$$Y_l = \left(Y_l^l, Y_l^{l-1}, Y_l^{l-2}, \ldots, Y_l^{-l} \right)^t$$

In this vector, for a given l, (2l-1) spherical harmonics are stored, and this functions span a rotationally invariant sub-space [ref 20 of the main text].

A set of vectors $\{v_i\}$ compose an invariant subspace V_s, under the transformation of a group $\{g_j\}$, if $g_j v_i \in V_S \quad \forall i,j$.

In the case of interest, we can therefore formulate:

$$Y_l(\theta+\theta_0, \varphi+\varphi_0) = o_l(\theta_0, \varphi_0) Y_l(\theta, \varphi)$$

where o_l is a unitary matrix representing the rotation group of transformations.

The rotations do not affect the norm of a vector:

$$\left\| Y_l(\theta+\theta_0, \varphi+\varphi_0) \right\| = \sqrt{\langle o_l Y_l | o_l Y_l \rangle} = \sqrt{\langle Y_l | o_l^+ o_l | Y_l \rangle} = \sqrt{\langle Y_l | 1 | Y_l \rangle} = \sqrt{\langle Y_l | Y_l \rangle} = \left\| Y_l(\theta, \varphi) \right\|$$

because o_l is a unitary matrix.

In other words the following relationship exists:

$$\mu_l = \left\| \begin{matrix} \left\langle f, Y_l^l(\theta+\theta_0, \varphi+\varphi_0 \right\rangle \\ \left\langle f, Y_l^{l-1}(\theta+\theta_0, \varphi+\varphi_0 \right\rangle \\ \dots \\ \left\langle f, Y_l^{-l}(\theta+\theta_0, \varphi+\varphi_0 \right\rangle \end{matrix} \right\| = \left\| \begin{matrix} \left\langle f, Y_l^l(\theta, \varphi \right\rangle \\ \left\langle f, Y_l^{l-1}(\theta, \varphi \right\rangle \\ \dots \\ \left\langle f, Y_l^{-l}(\theta, \varphi \right\rangle \end{matrix} \right\|$$

and the coefficients $\boldsymbol{\mu}_l$, independent on index m and rotationally invariant, can be obtained taking the norm of the descripted vectors.

(P → NP)		(NP → P)	
1bgx	3oay	1aj7	2e27
1fns	3q3g	1baf	2jb5
1g9m	3w9e	1c12	2ok0
1nmb	3zkm	1c5c	2xzc
1ob1	4aei	1cf8	2yk1
1ors	4f37	1ct8	2z92
1sy6	4fqi	1dl7	3cfd
1xiw	4lqf	1eap	3hns
2jel	4lu5	1fl6	3i9g
2w9e	4lvn	1h8s	3ls4
2zch	4m1g	1i7z	3ra7
3gi8	4m62	1i8m	3v0w
3grw	4nx3	1ind	43ca
3h3b	4ob5	1jgl	4c83
3ks0	4okv	1jnh	4hih
3nps	4tsa	1mex	4kzd
3o2d		1mh5	4m7j
		1n7m	4odt
		1q72	4odv
		1ub5	4ptu
		1um4	4s1d
		1uwg	4z8f
		1uz8	5cp3
		25c8	

Supplementary table 1

PDB IDs of antibodies incorrectly classified by the Method.

The columns (P → NP) and (NP → P) list the PDB IDs of protein binding antibodies classified as non-protein binding and non-protein binding classified as protein binding. Cases where the use of the actual number of contacts rather than the predicted number would have led to a correct classification are underlined.
